# Supplementary figures and images for: Uterus-specific transcriptional regulation underlies eggshell pigment production in Japanese quail
Source: PLoS One. 2022 Mar 10;17(3):e0265008. doi: 10.1371/journal.pone.0265008 (PMC8912178; doi:10.1371/journal.pone.0265008)

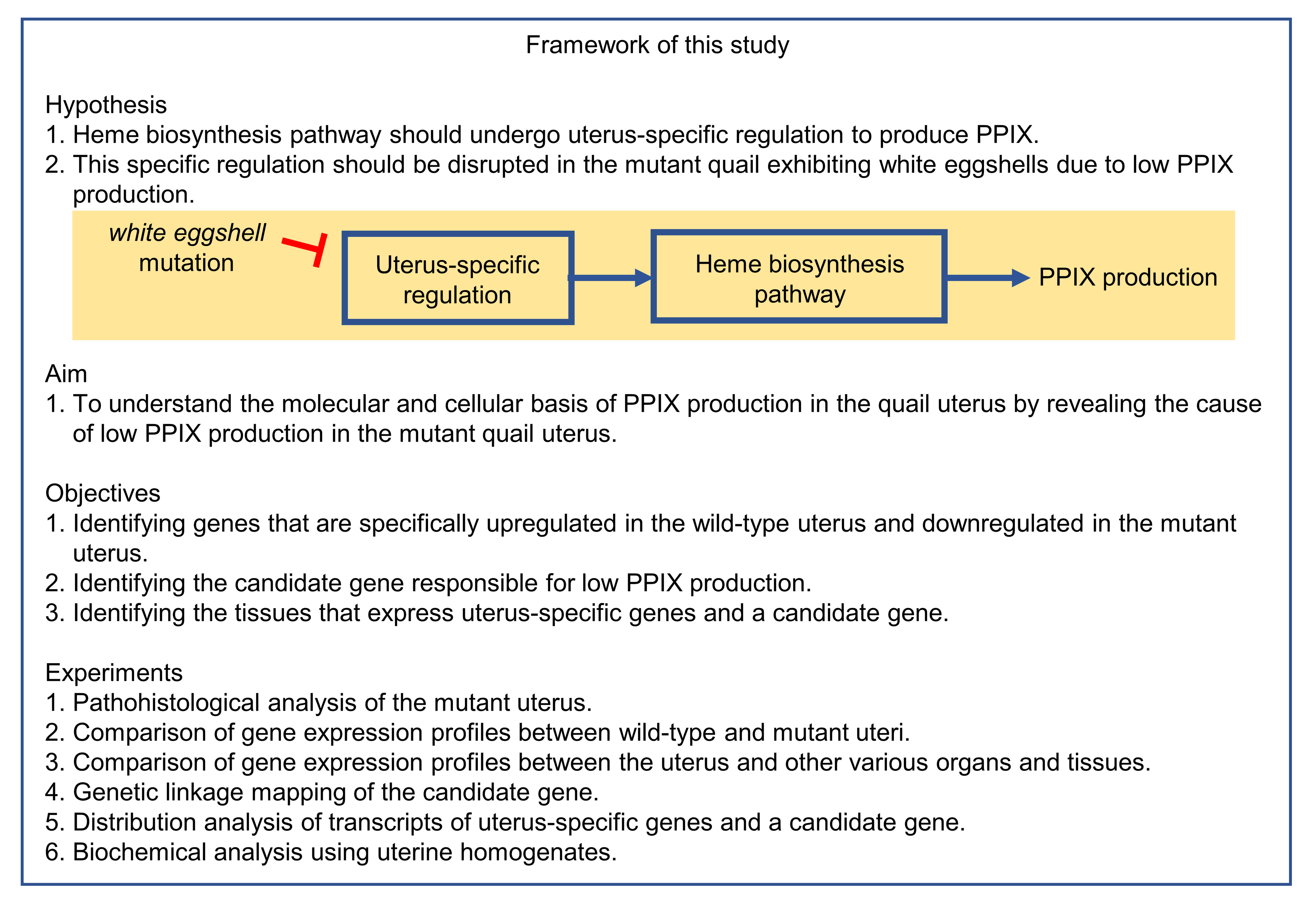

Supplement: S1 Fig — The image in the upper panel was prepared by modifying the figure from Sachar et al. (2016) [11]. (TIF) [file pone.0265008.s001.tif]

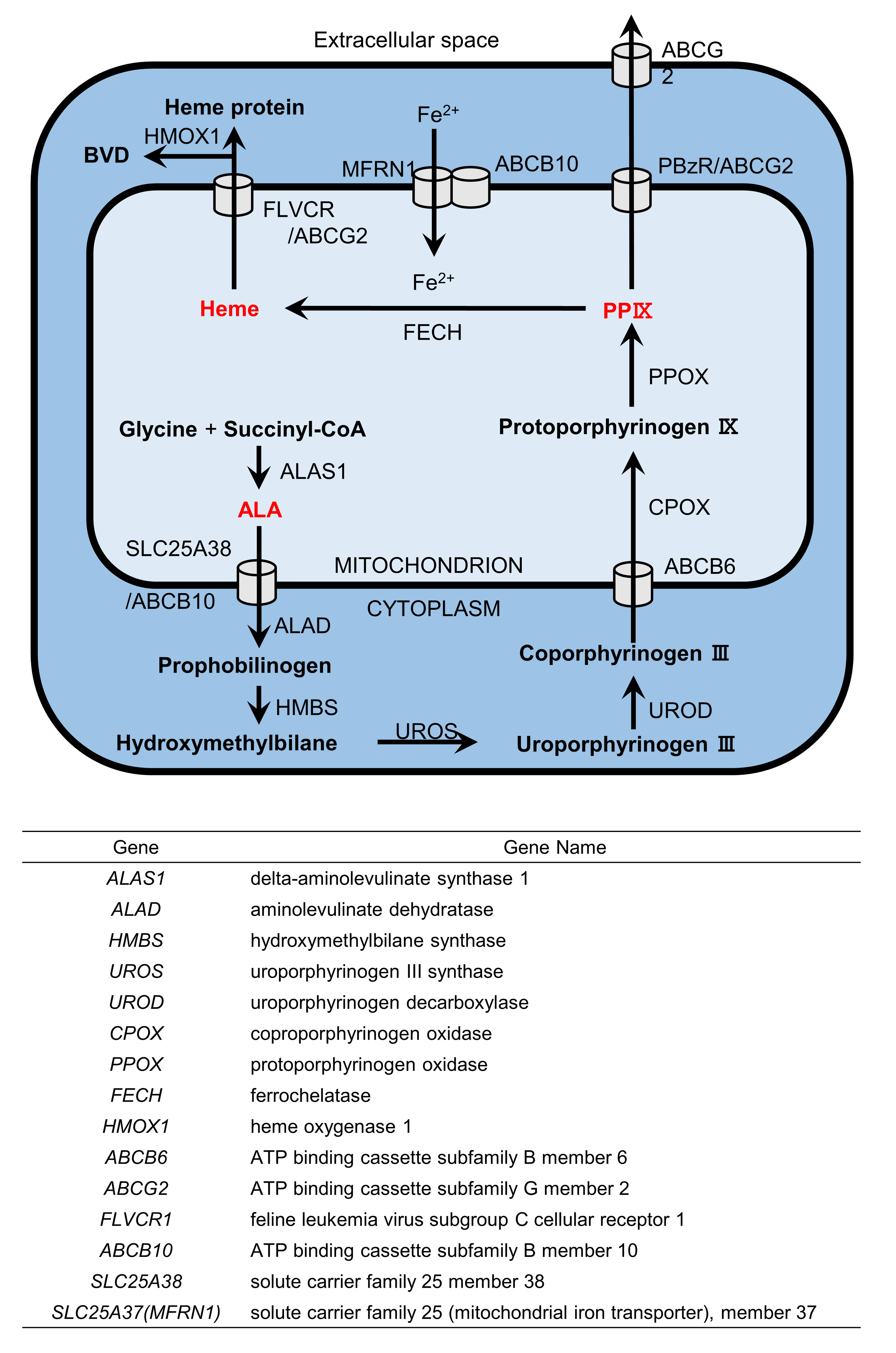

Supplement: S2 Fig — (TIF) [file pone.0265008.s002.tif]

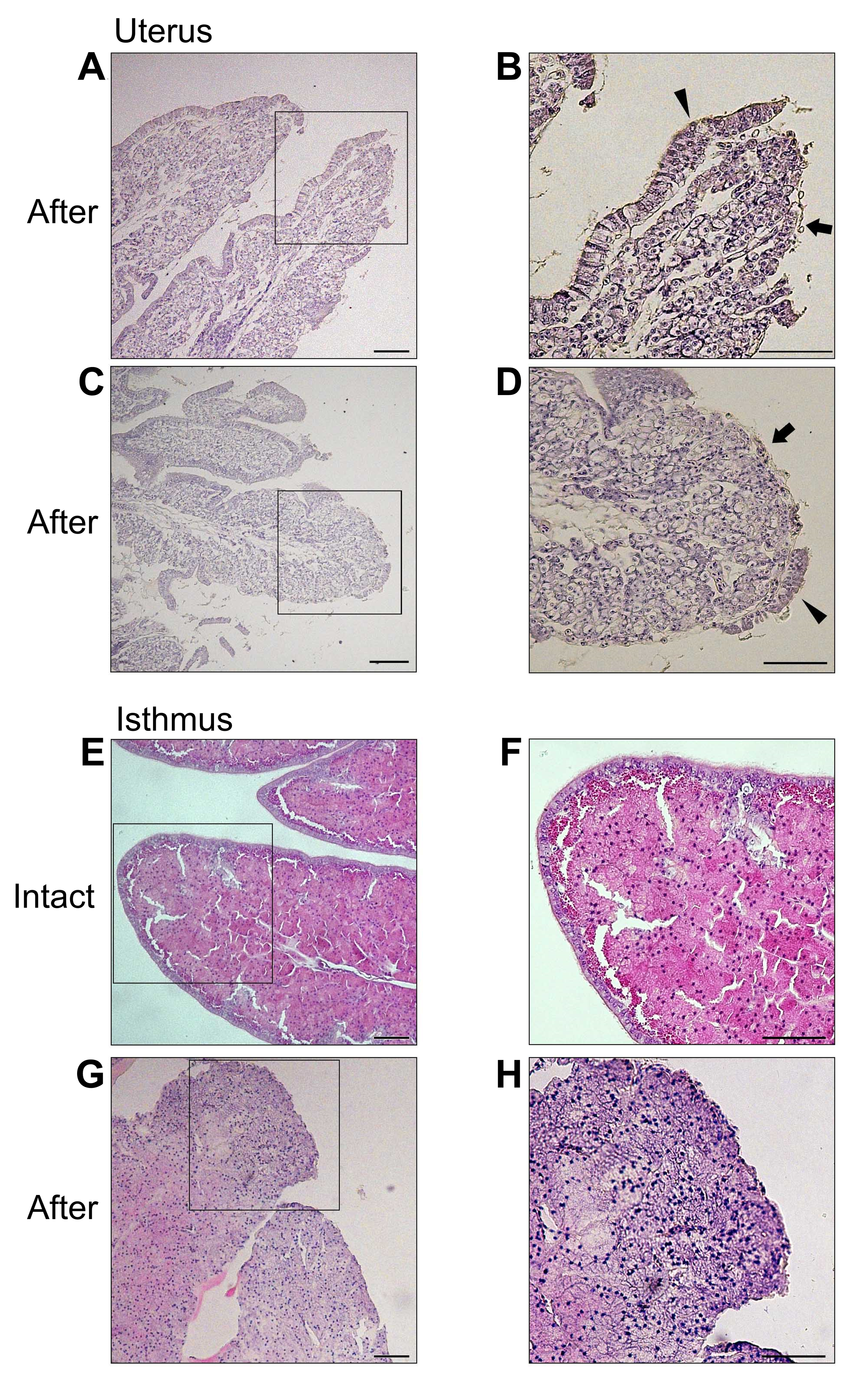

Supplement: S3 Fig — (A–D) Mucosae in uteri after brushing. (B, D) Higher-magnification images of the parts shown inside the frames in (A, C). The epithelium is removed from the mucosa (arrows). Arrowheads indicate the remaining epithelia. (E–H) Mucosae of the isthmuses before brushing (E, F) and after brushing (G, H). (F, H) Higher-magnification images of the parts shown inside the frames in (E, G). Scale bar indicates 100 μm. (TIF) [file pone.0265008.s003.tif]

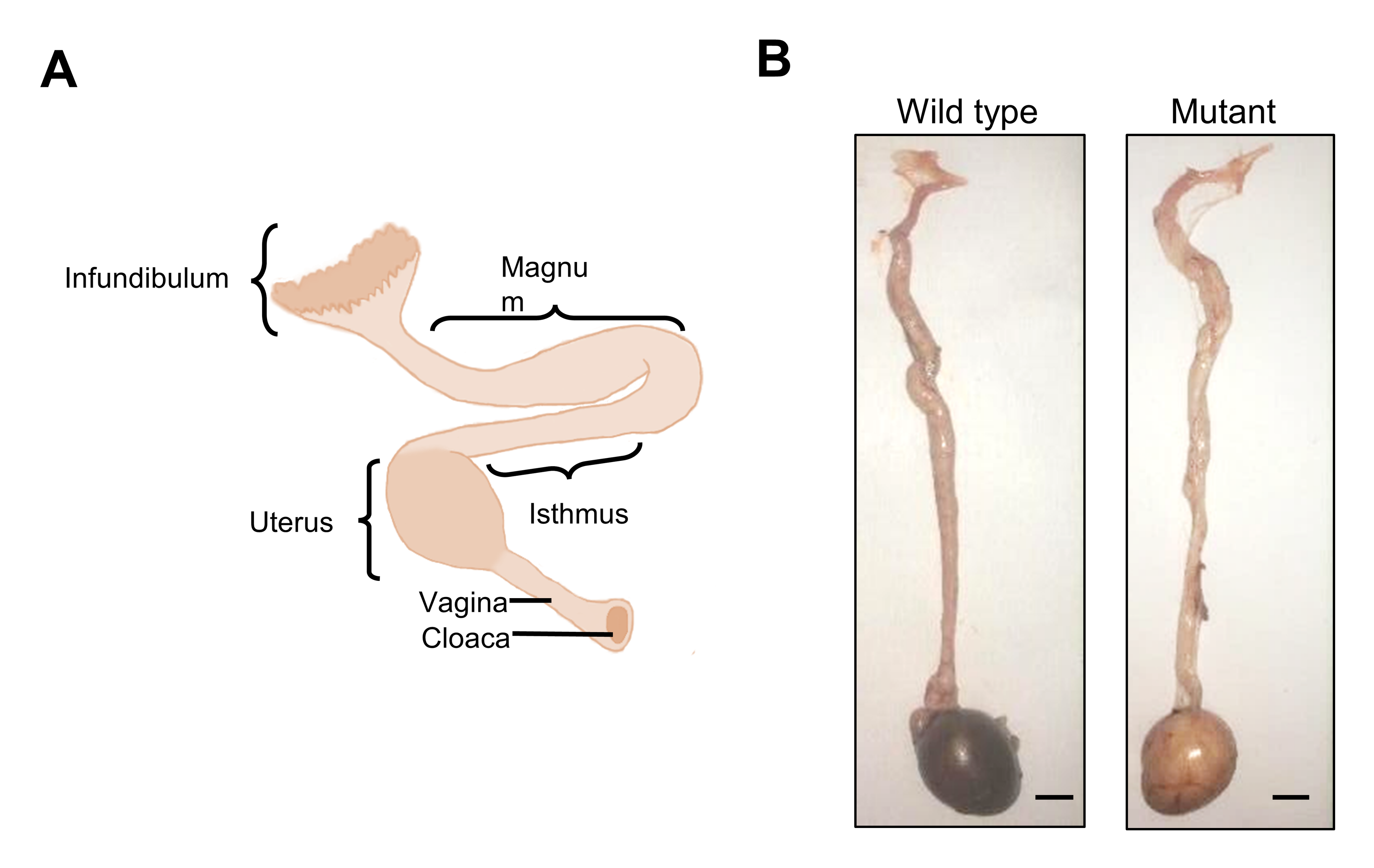

Supplement: S4 Fig — Schematic diagram of the quail oviducts containing an egg (A). The uteri of the wild-type and mutant quails were dark brown and pale brown, respectively (B). The vaginas were removed from the oviducts. Scale bar indicates 1 cm. (TIF) [file pone.0265008.s004.tif]

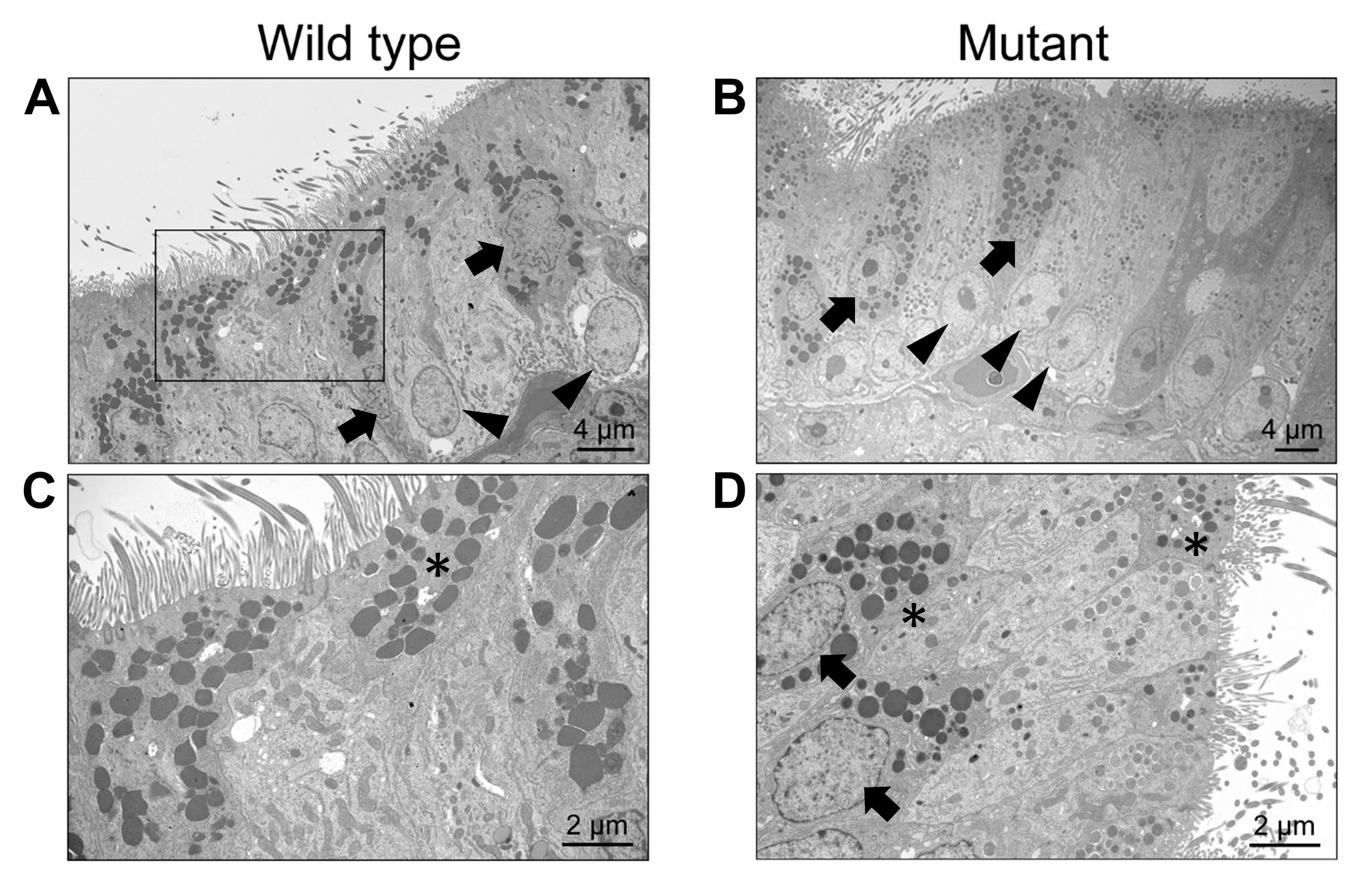

Supplement: S5 Fig — (A, B) Wild-type uterus. (C, D) Mutant uterus. The part shown inside the frame in (A) is enlarged in (C). Nuclei of apical and basal cells are indicated by arrows and arrowheads, respectively. Transport vesicles are observed as electron-dense granules in the apical cells of both the wild-type and mutant epithelia (asterisks). Magnifications are 4000× in (A), 10000× in (B), 1500× in (C), and 7000× in (D). (TIF) [file pone.0265008.s005.tif]

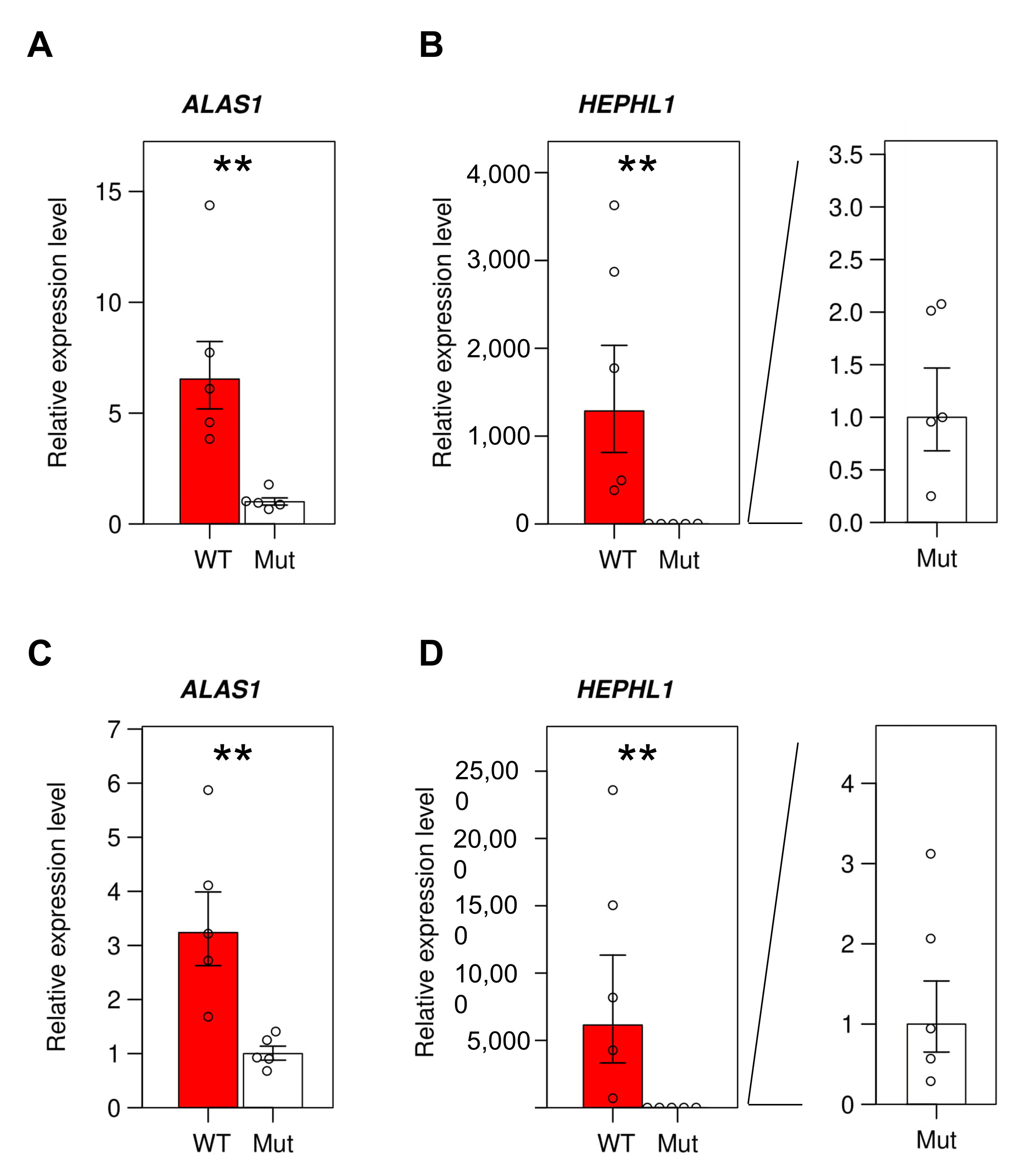

Supplement: S6 Fig — The graphs show the results of qPCR analysis using RNA samples (n = 5 for both wild-type and mutant quails) used for mRNA sequencing (A, B) and RNA samples (n = 5 for both wild-type and mutant quails) that were not used for mRNA sequencing (C, D). The expression levels in the wild-type uterus are shown as relative values to those in the mutant uterus, defined as 1. The expression level in each sample is shown as a circle. **p < 0.01. (TIF) [file pone.0265008.s006.tif]

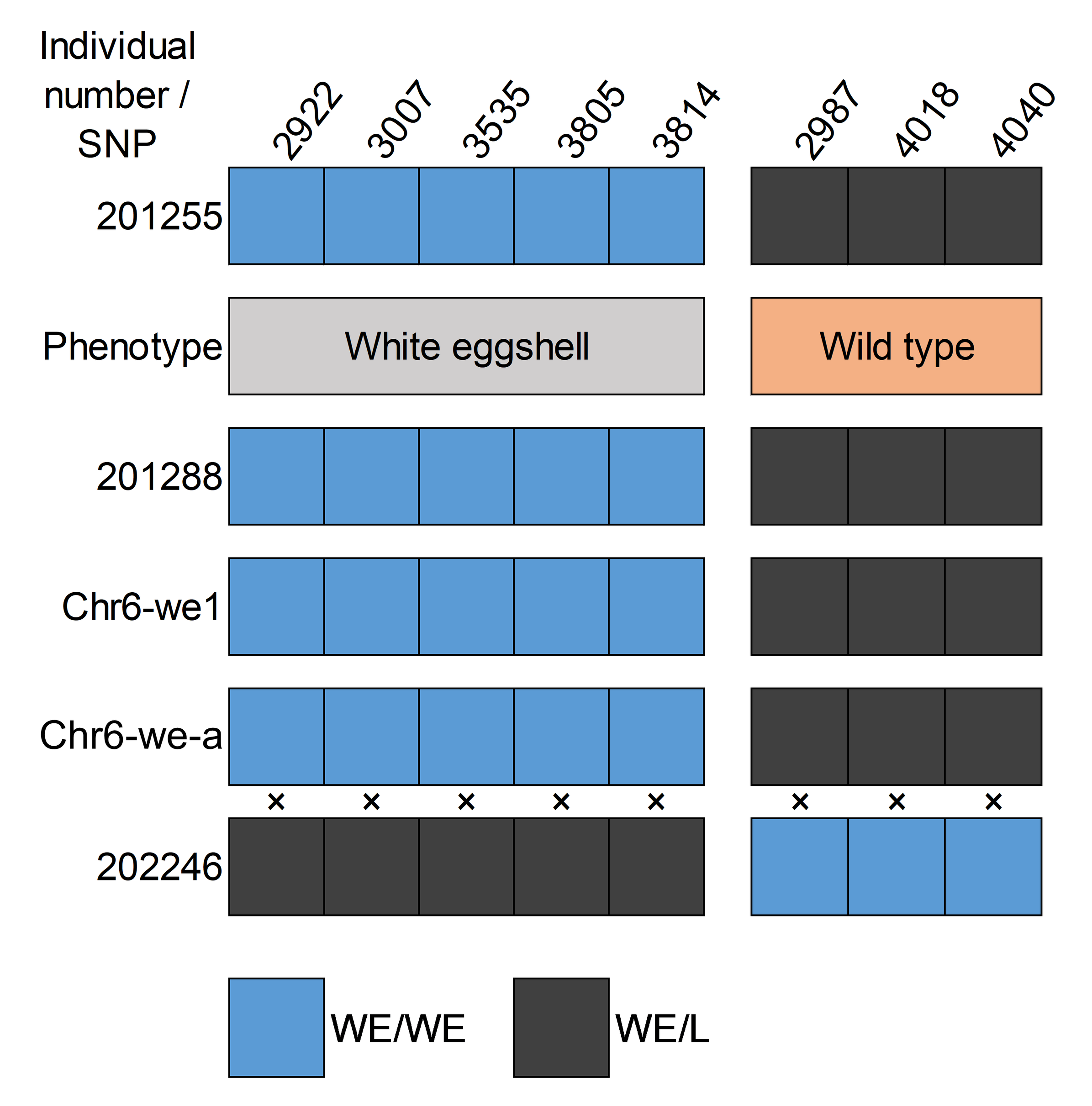

Supplement: S7 Fig — Genotypes of SNPs 201288, Chr6-we1, and Chr6-we-a were fully concordant with the eggshell color phenotypes. (TIF) [file pone.0265008.s007.tif]

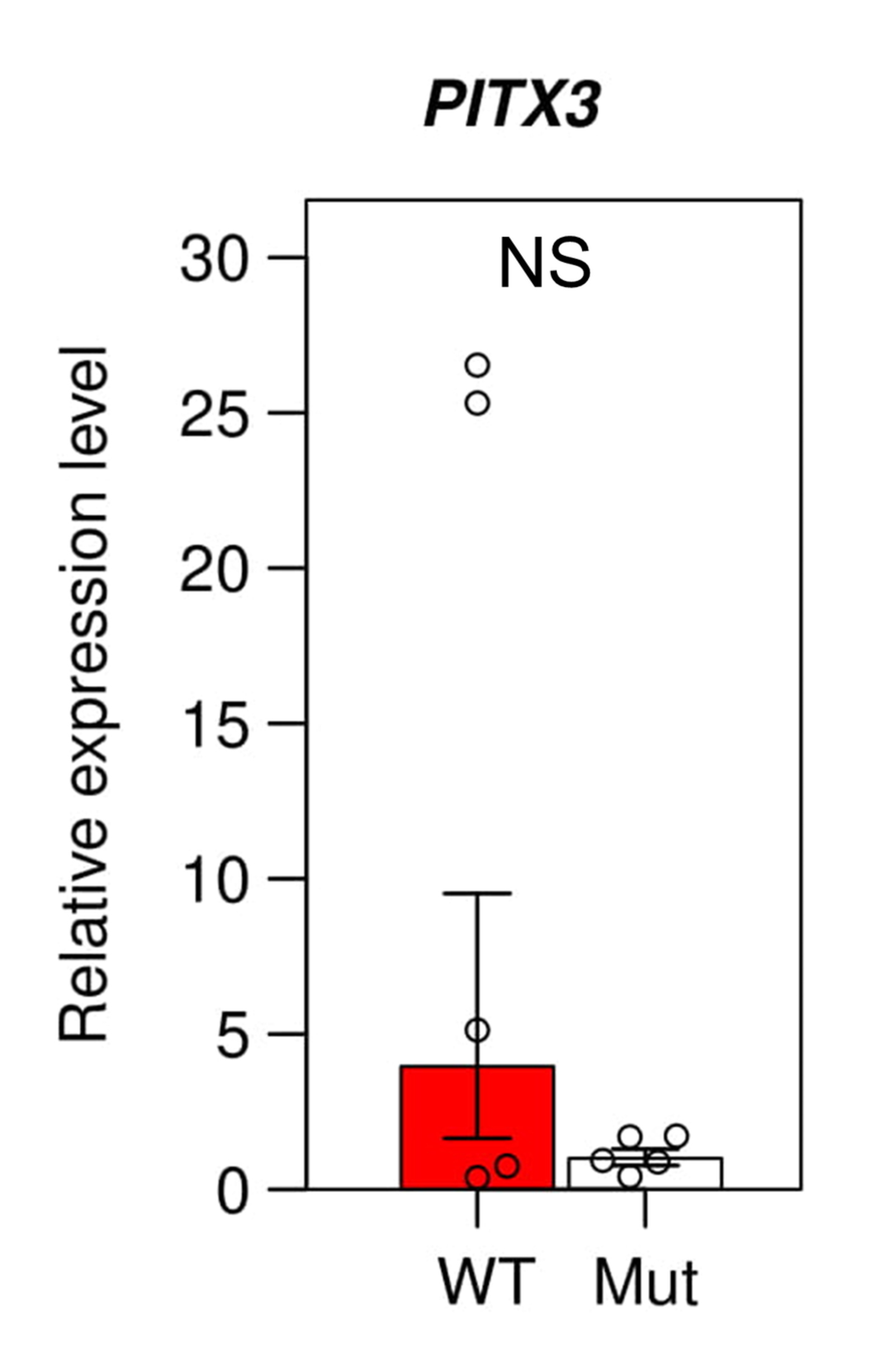

Supplement: S8 Fig — The graphs show the results of qPCR analysis using RNA samples (n = 5 for both wild-type and mutant quails) used for mRNA sequencing. The expression levels in the wild-type uterus are shown as relative values to those of the mutant uterus, defined as 1. The expression level in each sample is shown as a circle. NS, not significant. **p < 0.01. (TIF) [file pone.0265008.s008.tif]
