# Supplementary material for: Uterus-specific transcriptional regulation underlies eggshell pigment production in Japanese quail
Source: PLoS One. 2022 Mar 10;17(3):e0265008. doi: 10.1371/journal.pone.0265008 (PMC8912178; doi:10.1371/journal.pone.0265008)
Supplement: S2 Data — (DOCX) [file pone.0265008.s010.docx]

**Sequence of *ALAS1* cDNA used for *in situ* hybridization (nt): (1,298 bp)**

5′- CATACCTACAGAGTGTTCAAAACGGTGAACCGAAAGGCACAGATCTTTCCCATGGCAGATGACTACTCTGACTCCCTGATCACCAAGAAGGAGGTGTCTGTCTGGTGCAGCAATGATTACCTGGGCATGAGTCGTCACCCTCGTGTGTGCGGAGCAGTGATGGATACACTGAAACAACATGGTGCTGGAGCAGGAGGCACAAGAAATATTTCAGGAACAAGCAAATTTCACGTTGACTTGGAAAAAGAACTGGCTGATCTTCATGGAAAAGATGCAGCCTTGTTGTTCTCATCTTGCTTTGTAGCCAATGATTCCACCCTCTTCACTCTTGCTAAAATGCTGCCAGGTTGTGAGATCTACTCTGATTCTGGAAACCATGCCTCCATGATCCAGGGCATTCGAAACAGCAGGGTGCCGAAACACATCTTTCGCCATAACGATGTCAACCATCTTCGAGAGCTATTGAAGAAGTCTGATCCGTCTACCCCTAAAATTGTTGCATTTGAAACTGTGCATTCTATGGATGGTGCTGTCTGCCCTCTGGAAGAGCTGTGTGACGTGGCTCACGAGCACGGGGCAATCACTTTTGTGGATGAAGTGCACGCTGTGGGGCTGTATGGAGCTCGAGGTGGTGGGATAGGGGACCGCGATGGAGTCATGCACAAGATGGACATCATCTCTGGAACTCTTGGCAAGGCCTTTGGCTGTGTGGGTGGATACATCTCCAGTACAAGTGCTCTGATAGACACTGTCCGTTCATATGCTGCTGGTTTCATTTTCACTACATCCCTGCCACCCATGCTCCTGGCTGGTGCCCTCGAATCTGTCCGAACTCTGAAAAGTGCTGAGGGACAAATCTTGAGGCGCCAGCACCAACGCAATGTGAAGCTTATGAGACAGATGCTGATGGACGCAGGGCTTCCTGTAGTACACTGCCCAAGTCACATCATTCCAATAAGGGTTGCAGATGCTGCTAAAAATACAGAGATCTGTGACAAGCTGATGAGCCAGCACAGCATCTATGTCCAAGCCATCAACTACCCCACAGTTCCTCGTGGAGAAGAGCTGCTACGTATTGCTCCTACACCTCACCACACCCCTCAGATGATGAGTTATTTCCTTGAAAAGCTGCTGGCTACATGGAAGGATGTTGGGCTGGAGCTGAAGCCTCACTCATCAGCTGAATGCAACTTCTGCAGAAGACCTCTCCACTTCGAAGTGATGAGTGAAAGGGAAAGATCCTACTTCAGTGGCATGAGCAAACTAGTATCTGTCAGTGCATGAAAGTAATGGTGTTC-3′.

**Sequence of *HEPHL1* cDNA used for *in situ* hybridization (nt): (1,434 bp)**

5′-AAACACCTACAGATGGAATGTCCCAGAGCAATCAGGCCCTGGGAAAACAGATCCCAACTGTATCACTTGGGTTTACTATTCAACAGCAAATTTCGTCAAGGACACGTATAGCGGTCTGATTGGTCCCCTTGTAGTCTGCAGAAAAGGAGTTCTAGATGAAAATGGCCAGAGAAAAGACATTGATCGTAAATTTACTCTTCTGTTTATGGTGTTCGATGAAAACAAATCCTGGTATTTGGAAGAGAACATTGAAACATACCTTCACAAGAGTCCCGATGATTTTAATTCCACTAAGAACTTTGTAGAAGGCAACAGCAAGCATGCCATCAATGGGAAGATTTATAACAGTCTCCTGGGTCTAACCATGAATGAAGGGGATACGACAAACTGGTATTTGATAGGAACGGGTAATGAAGTAGATGTGCATACAGTCCATTTCCATGCACAGACCTTCATCTTCAAGACAGATAAAGACCACAGAGGAGATGTATATGACCTTTTCCCTGGGACTTTCCAAGCTGTTGAACTTGTAGCGGAAAACCCTGGAACGTGGCTTCTGCACTGCCACGTAGCTGACCACATACACGCTGGCATGGAAACGACCTACACCAACAATAAATCAGAGCTGGAAGCCCCTTCAGAAGGAGGACTGACGACCACCACAGCTTATGGTACAACTACCGCACACAACAGGACCACTGCAAAGGATGCTGACAGCCAAGGGGACAACGTCGGCTCTCCATGACAAAAGTGCACTTCTAGCAGATTCCCTGTAAATCCCCACTTTCCATAGAGCCATGCCGGGTTTCACCAAATGCTGCTGCTACAACTAGAGCAACACACACTTCAGCATTCCCATCCCATTGCCAGCACTCCTGAGAGCTGCAGGAAAGTGCTTCAGGAGCCACGTATCACCCATTGCCATTCCCACCCCACCACCACTCAGCTGTGCCCAAGGCACCACACCACTTGGCCAGCATTCTCATGGAGCAGACAGGATTGCACCCATGTGCTCTATGCAGCACCATGGAGTGCACACTGCTTTAGCATCCAGCTTCTGAAGGCACAGGTGTAAGTCTGGAAGTTCTTAAGGTTTCCCAGAGAGAAGCCAGCCTTACACCAGAGTCTGCATCAATGAATTCACCAGCATTTAACCGATTTTTCCCTTTTTTTGGTTCTCTTCACCCTGTTAAATGAGGAAGCTGTGCACTGGGGACAGATTTTTAAAGGCATTTAAGTAGCCACCAATATACTTCAGCACCTGATGAAGCTCAGCATCTGCAACAGGTTTGTTAATGCCTTTAAAAATCTAGCCCTGAGCAGCCTCAATGAAGTTATACTCTGTGATTGCTGCTTGTTGTGGTCCTGATTCTGCAACTCTTCTCTGCACTGAGCAGTGCTTTGTTCCCAAGCGTTAATTGGGCTGCTGATATC-3′

**Sequence of *PITX3* cDNA used for *in situ* hybridization (nt): (1,101 bp)**

5′- ATTCCTGCCCCATGGATTTCAACCTGCTGGCGGACGCGGAGGCTCGCAGCCCAGCCCTGTCCCTCTCAGACTCTGGCACCCCCCAGCACGAGCACAGCTGCAAGGGGCAGGACCATAGCGATACTGAGAAGTCCCAGCAGAACCAGACAGACGACTCCAACCCCGAGGACGGCTCTCTCAAGAAGAAGCAGCGGAGGCAGCGGACGCACTTCACCAGCCAGCAGCTCCAGGAGCTGGAAGCCACGTTCCAGAGGAACCGCTATCCTGACATGAGCACCAGGGAGGAGATTGCAGTCTGGACCAACCTGACGGAGGCACGAGTGCGGGTCTGGTTCAAAAATCGCAGGGCTAAGTGGAGGAAACGGGAGAGGAACCAGCAGGCCGAGCTCTGCAAGAACAGCTTTGGAGCCCAGTTCAATGGACTGATGCAGCCCTATGATGACGTGTATTCCAGCTATTCCTACAACAACTGGGCCACCAAAGGGCTTGCCACCAGCCCGCTCTCAGCTAAAAGCTTCCCATTCTTCAACTCCATGAACGTCAGCCCCCTCTCCTCCCAGCCCATGTTCTCCCCACCCAGCTCCATCGCCTCGATGACCATGCCTTCATCCATGGTCCCTTCTGCAGTGACTGGCGTCCCGGCCTCCAGCCTCAACAACCTGGGAAACATCAACAACCTGAACAGCCCAAGCCTCAACTCTGCCGTCTCATCCAGTGCCTGTCCTTACGCCTCCACAGCCAGCCCCTACATGTACAGGGACACGTGCAACTCCAGCCTGGCAAGTCTGAGGTTGAAGGCCAAGCAGCATGCCAACTTTACTTACCCGGCGGTGCAGACGGCAGCTTCCAACCTAAGCCTTGCCAATACGCCGTGGACAGGCCTGTATGAAGTGCTGCTCTCTGCTGACTGGGGACTTGACCTTAGCCTGACACAAGGAGACCTTTAGCCCTACAACAGTGTACATCCTGCAGAGCAAGAGTGGAAGCAAGAGAGAGCAAAAGAGCATGAGAAAGTGAGGGCACACAGCAAGCAGGCAGATGGACCTCGATGAAGATTTGTCTAACTATCGTATGGTGAATTTTGACTGTCTTTCCCCTCCC-3′.
