# Supplementary material for: Uterus-specific transcriptional regulation underlies eggshell pigment production in Japanese quail
Source: PLoS One. 2022 Mar 10;17(3):e0265008. doi: 10.1371/journal.pone.0265008 (PMC8912178; doi:10.1371/journal.pone.0265008)
Supplement: Attachment — Submitted filename: response letter 220215.docx [file pone.0265008.s025.docx]

**Response to Reviewer #2**

We wish to express our appreciation to the Reviewer for his or her insightful comments, which have helped us significantly improve the paper.

1) *“Authors must concentrate on the formatting, and use of symbols, etc., throughout the manuscript.”, “Proper formatting needed in the materials and methods, results, discussion and conclusion section.”*

Response:

We have revised the formatting properly for readability. The descriptions in the Materials and methods, results, and discussion sections have been arranged. Please see the revised manuscript with track changes for details. Main revisions are as follows.

Lines 140. “The average number of mapped read pairs is also shown in S1 Data.”, which was placed in the result section in the original manuscript, has been moved to the Materials and Methods section, for readability.

Lines 250−256. “For the synthesis of RNA⎯ *paired-like homeodomain 3* (*PITX3*))”, which was placed at the end of this paragraph, has been moved next to the first sentence of the paragraph for readability.

Line 469. The line break position has been changed. In the original manuscript, it was placed before “The 172 kb candidate region harbored⎯” (line 443 in the revised manuscript).

Lines 566−591. Discussion about the candidate gene and the hypothetical model has been moved from the 4th and 5th paragraphs in the original manuscript to the 3rd and 4th paragraphs in the revised manuscript.

Lines 603−608. The discussion about *in situ* hybridization analysis has been moved from the 3rd paragraph to the 5th paragraph.

Consequently, the numbering of reference has been changed as follows.

#53 in the original manuscript > #55 in the revised manuscript

#54 > #53

#55 > #54

The reference list has been changed along with these alterations.

The result of biochemical assay and the hypothetical model of the genetic cascade in the original Fig 6 have been divided into Fig 6 and Fig 7 in the revised manuscript, for readability.

We have defined “uterus-specific genes” and “the candidate region” as follows for readability.

Lines 106−110.

“To identify the differentially expressed genes (DEGs) between the wild-type and mutant uteri and detect the genes that are specifically upregulated in the uterus (uterus-specific genes) compared to the other six organs (heart, liver, intestine, kidneys, lungs, and brain) and two tissues (muscle and isthmus), we performed mRNA sequencing of the uteri and isthmuses from the wild-type and mutant quail.”

Line 193−195.

“sequence variants that were found in the candidate causative region (candidate region) were annotated using SnpEff v4.3t [33].”

In the author contribution section (at the end of the manuscript), the short names of several authors have been changed, as follows.

Lines 917−923.

Ishishita > Is

Iwasaki > Iw

Ohkawa > Ohk

Ohmori > Ohm

We have arranged or added several subtitles in Materials and Methods and Result sections for readability, as follows.

Line 105. “mRNA sequencing” has been change to “mRNA sequencing and differential gene expression analysis”.

Line 152−153. “Double-digest restriction site-associated DNA (ddRAD) sequencing and association test” has been changed to “Construction of a reference family, double-digest restriction site-associated DNA (ddRAD) sequencing, and association test”.

Line 232. “Transmission electron microscopy” has been changed to “Transmission electron microscopy (TEM)”.

Line 286. “Biochemical assay for the PPIX-forming ability of the uterus” has been changed to “Biochemical assay to evaluate the PPIX-forming ability of the uterus”.

Line 323−324. “Fig 1. The *white eggshell* phenotype” has been changed to “Fig 1. White eggshell phenotype and low accumulation of brown pigments in the uterine mucosa of the mutant quail.” for scientific accuracy.

Line 342−343. “Characterization of the mutant uterus by gene expression analysis” has been changed to “Characterization of the mutant uterus by gene expression analysis and identification of uterus-specific genes”.

Line 356−357. “Fig 2. Genes that are specifically upregulated in the wild-type uterus and downregulated in the mutant uterus.” has been changed to “Fig 2. Identification of genes that are specifically upregulated in the wild-type uterus and downregulated in the mutant uterus.”.

Line 386. “Genetic linkage mapping of the candidate region responsible for the white eggshell phenotype” has been changed to “Genetic linkage mapping of the responsible locus”.

Line 404. “Fig 3. The candidate region of the *white eggshell* (*we*) phenotype is mapped to chromosome 6.” has been changed to “Fig 3. Genetic linkage mapping of the *we* locus.”.

Line 440−441. “Identification of the causative mutation and causative gene around the we locus” has been added.

Lines 494−495. “Distribution analysis of *PITX3*, *ALAS1*, and *HEPHL1* transcripts in the uterine mucosa” has been added.

2) *” Framework figure is required.”*

Response:

We have added framework of this study in S2 Fig, therefore,

Line 77−78. We have mentioned “The framework of this study in shown in S2 Fig.” at the end of Introduction.

Line 814. We have added a figure caption “S2 Fig. Framework of this study.”.

The numbering of the remaining Supporting Figures has also been changed.

3) *”In materials and method section: authors should explain why each item of methodology was done.”*

Response:

Lines 91−92, 106−110, 154−157, 181−183, 224, 233−235, 249−250, 277−278. We have described the objective of each item at the beginning of paragraphs. Please read the revised manuscript with track changes for details.

We have also mentioned objectives of experiments in the result section at lines 343−345, 389, 442, and 496.

4) *”Real Time-PCR's raw data in the supplementary for scrutiny.”*

Response:

As suggested, we have added raw data of Real Time PCRs as S6−S11, S14, and S15 Data.

Because we have added these data, numbering of Supporting Data and has been changed and captions in pages 39−41 have been changed.

5) *”In conclusion section authors should provide some future prospectus related to present study.”*

Response:

We have added the following sentences at the end of the conclusion.

Lines 635−640. “In future studies, we should identify the causative gene and mutation responsible for white eggshells and investigate the functional roles and transcriptional mechanisms of the 11 genes that were specifically expressed in the wild-type uterus and downregulated in the mutant uterus. In addition, the specific roles of apical and LP cells in PPIX production should be further elucidated.”

6) *Other revisions*

Line 40. “*5 aminolevulinic acid synthase 1*” has been corrected to “*5'-aminolevulinate synthase 1* ”, for scientific integrity.

Line 93, “female quail strains” in the original manuscript has been changed to “quail strains”, to inform accurately.

Lines 196−197. “informative SNP markers that could reveal the parental origins of chromosomal regions of the parental quail.” has been changed to “informative SNP markers that could reveal the parental origins of chromosomal regions of the F_2_ progeny.” to inform accurately.

Lines 374−376.

We have mentioned the result of comparison of heme biosynthesis-related genes’ expression between the uterus and other organs and tissues for scientific integrity, as follows. “The expression levels of genes related to heme and iron metabolism in the wild-type uterus were compared to those in other organs and tissues (S3 Table).”. New Supporting Table (S3 Table) has been added.

Lines 381−384. We have added the result of GO term enrichment analysis of the 261 DEGs and associated Table (as S6 Table), for scientific integrity.

The following careless mistakes have been corrected as follows.

Line 431.

Fig 3E (at line 452 in the original manuscript) > Fig 3F

In Fig 2C.

The number of DEG (down) in the circle has been corrected from 250 (all 261 DEGs – 11 overlapping genes) to 102 (=113 downregulated genes – 11 overlapping genes).

Because we have added S3 and S6 Tables, numbering of Supporting Tables has been changed and relevant captions in Page 38−39 have been changed.

In addition, the following description at lines 333−335 in the original manuscript,

“The results of GO term enrichment analyses of the uterus-specific upregulated genes are shown in S4 Table. It is of interest to note that many uterus-specific upregulated genes were related to GO “biological process” (GO:BP) terms, such as cellular ion homeostasis and ion transmembrane transport. This may be related to eggshell calcification in the uterus.”

has been removed, and the following description has been added at lines 381−384 in the revised manuscript.

“The results of GO term enrichment analyses of the uterus-specific upregulated genes and the DEGs are shown in S5 and S6 Tables, respectively. GO “biological process” terms, such as extracellular matrix organization and cation homeostasis, were overrepresented in both groups”

Line 389. “we performed chromosomal mapping of the mutation by genetic linkage analysis” has been changed to “we performed chromosomal mapping of the locus by genetic linkage analysis”.

Lines 461, 476, 477, 631.

“the candidate region” has been changed to “the 172 kb candidate region” to inform accurately.

Lines 577−580.

We mentioned “Epigenetic approaches, such as chromatin immunoprecipitation sequencing with anti-H3K27ac antibody [53] or assay for transposase-accessible chromatin using sequencing (ATAC-seq) [54], to compare active regulatory regions in uterine cells between the wild type and mutant would be useful for identifying a candidate as the uterus-specific enhancer of *PITX3*.” in the original manuscript. To inform accurately, we have changed the underlined part to “identifying candidates of uterus-specific enhancers of *PITX3*”.

Lines 624−625. We have changed from “we performed transcriptome analysis of wild-type and mutant uteri and genetic linkage mapping of the causative mutation responsible for white eggshells.” to “we performed transcriptome analysis of wild-type and mutant uteri and genetic linkage mapping of the genomic region responsible for white eggshells.”

Lines 653−657. Data Availability statement has been moved from the Materials and Methods section. We mentioned “Other miscellaneous information is available from the corresponding author upon request.” in the original manuscript. However, we have removed this sentence, because all data is available from Supporting Information, public database, or github.com.

Many minor corrections in terms of grammatical accuracy have been made though English proofreading. See the revised manuscript with track changes for details.
